# Psychometric properties of the stress control mindset measure in university students from Australia and the UK

**DOI:** 10.1002/brb3.1963

**Published:** 2020-11-24

**Authors:** Jacob J. Keech, Sheina Orbell, Martin S. Hagger, Frances V. O’Callaghan, Kyra Hamilton

**Affiliations:** ^1^ School of Health and Behavioural Sciences University of the Sunshine Coast Sippy Downs Australia; ^2^ School of Applied Psychology Menzies Health Institute Queensland Griffith University Brisbane Australia; ^3^ Department of Psychology University of Essex Colchester UK; ^4^ University of California Merced CA USA; ^5^ Faculty of Sport and Health Sciences University of Jyväskylä Jyväskylä Finland

**Keywords:** coping, implicit theories, mindset, stress, stress beliefs

## Abstract

**Introduction:**

Beliefs about the consequences of stress, stress mindsets, are associated with health and performance outcomes under stress. This article reports the development and examination of the psychometric properties of a measure of stress mindset: The Stress Control Mindset Measure (SCMM). The measure is consistent with theory on mindsets about self‐attributes and conceptualizes stress mindset as the extent to which individuals endorse beliefs that stress can be enhancing.

**Methods:**

The study adopted a correlational cross‐sectional survey design in two student samples. Undergraduate students from an Australian university (Sample 1, *N* = 218) and a UK university (Sample 2, *N* = 214) completed the SCMM and measures of health and well‐being outcomes.

**Results:**

Confirmatory factor analyses supported a four‐factor structure and strict measurement invariance across samples (ΔCFI < 0.01). Reliability, convergent validity, discriminant validity, and concurrent validity of the overall SCMM were supported in both samples. Incremental validity was supported for most outcomes, accounting for significantly more variance (between 2.2% and 5.9%) in health and well‐being outcomes than an existing measure.

**Conclusions:**

Current data provide preliminary support for the SCMM as a reliable and valid measure with good psychometric properties and theoretically consistent relations with health outcomes under stress. Findings provide initial evidence supporting the potential utility of the SCMM in future research examining relations between stress mindsets and health and performance outcomes.

## Graphical abstract

This article reports the development and examination of the psychometric properties of a measure of stress mindset: The Stress Control Mindset Measure (SCMM). The measure conceptualizes stress mindset as the extent to which individuals endorse beliefs that stress can be enhancing. Findings provide evidence supporting the validity and reliability of the SCMM and its utility for use in future research examining relations between stress mindsets and health.

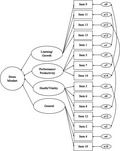

## 1 INTRODUCTION

Stress is defined as the tension experienced when one perceives an external event to outweigh their capacity to cope afforded by their coping resources (Lazarus & Launier, 1978; Lovallo, 2015). University students in developed nations such as the US and Australia often report high levels of stress (American College Health Association, 2017; Casey, 2014). While common guidance is that stress must be reduced or removed (Crum et al., 2013), barriers to using time‐consuming strategies such as relaxation exist in demand‐intensive environments. However, emerging evidence has highlighted the potential for positive stress‐related outcomes. Recent research has found that beliefs about the consequences of stress itself may be influential in determining the adaptiveness of the stress response (Crum et al., 2013; Keller et al., 2012; Nabi et al., 2013). Crum and colleagues (2013), for example, have found that holding a *stress‐is‐enhancing mindset*—the belief that stress results in increased performance and productivity, health and well‐being, and learning and growth—is associated with favorable self‐reported outcomes such as lower perceived stress and health symptoms. This includes increased work performance, more adaptive cortisol reactivity profiles, and greater desire for feedback under acute stress. At the other end of the spectrum is a *stress‐is‐debilitating mindset*, which is the belief that stress results in reduced productivity, health/well‐being, learning, and growth. Stress mindset has also been found to be related to, but distinct from, other stress‐related variables such as amount of stress, stress appraisal, coping skills, and social support (Crum et al., 2013). The distinction between beliefs that stress‐is‐debilitating and stress‐is‐enhancing are conceptualized as a spectrum that people can be placed on based on measurement of stress mindset. When manipulating stress mindset in prior research, the goal has been to increase the extent to which an individual endorses a stress‐is‐enhancing mindset (Crum et al., 2013, 2017).

At face value, the idea that holding a stress‐is‐enhancing mindset leads to more adaptive outcomes draws parallels with challenge versus threat appraisals in the transactional model (Kilby & Sherman, 2016). In this model, challenge appraisals of stressful stimuli lead to more adaptive outcomes (Lazarus & Folkman, 1984). Bivariate correlations reported by Kilby and Sherman (2016) show that stress mindset is positively related to challenge appraisals and negatively related to threat appraisals. However, the associations were small, indicating that stress mindset and appraisal are unique constructs. The key difference is that appraisals relate to stressor‐specific evaluations, whereas stress mindset refers to beliefs about the consequences of stress and are theorized to apply across stressors and situations (Crum et al., 2013).

Experimental and correlational studies have demonstrated effects of stress mindset on psychological and physical well‐being, coping behaviors, and affective outcomes among those experiencing stress. For example, one experimental study observed an effect of a stress mindset manipulation on depression and anxiety symptoms from baseline to three days post‐intervention among financial company employees (Crum et al., 2013). Another experimental study using a manipulation evoking a stress‐is‐enhancing mindset was found to increase cognitive flexibility, attention toward positive stimuli, positive affect, and dehydroepiandrosterone sulfate secretion (DHEAS; the anabolic “growth” counterpart of cortisol) for both challenging‐ and threatening‐appraised stressors (Crum et al., 2017). Correlational studies have revealed associations between stress mindset and improved coping behaviors, greater perceived physical and psychological well‐being, and better academic performance when experiencing ecological stressors (Casper et al., 2017; Keech, Hagger, O’Callaghan, & Hamilton, 2018). Together, these findings provide consistent support for the premise that stress mindset can assist in identifying the mechanisms by which stress influences health, well‐being, and performance. Further, the ability of stress mindset to be manipulated via relatively brief and simple interventions suggests that they may be useful for application in non‐clinical interventions aimed at effective stress management as advocated elsewhere (e.g., Hagger, Keech, & Hamilton, 2020; Keech et al., 2020a,2020b), which is particularly important given that just 13% of Australians report seeking professional support in dealing with stress (Casey, 2014).

### 1.1 Current divergence in theory and measurement of stress mindset

Crum et al. (2013) outlined evidence regarding both debilitating and enhancing consequences of stress, suggesting that consideration of the enhancing consequences of stress is often neglected. They emphasized the value of a more “nuanced view of stress that recognizes that while experiencing stress can debilitate health and performance, stress can also fundamentally enhance health and performance” (p. 717). Crum and colleagues (2013) also describe their results with the caveat that not all stress is enhancing, but rather that it can be utilized to be enhancing. This conceptualization of stress mindset is consistent with extant theory regarding mindsets about self‐attributes, which describes mindsets as the extent to which an individual believes a fundamental attribute such as intelligence, personality, or willpower to be malleable or incremental, as opposed to being fixed or stable (Dweck, Chiu, & Hong, 1995; Dweck & Yeager, 2019; Job, Dweck, & Walton, 2010; Yeager & Dweck, 2012). For example, an individual may hold the mindset that intelligence is incremental and can increase or decrease, which contrasts with holding the mindset that intelligence is fixed and cannot be changed. The idea that stress can be “utilized” adaptively by an individual is also consistent with extant theory regarding mindsets about self‐attributes, where the role of the individual in influencing the malleable attribute is emphasized. For example, Blackwell et al. (2007) report that individuals endorsing an incremental mindset about intelligence employ strategies such as working harder in the face of setbacks and they consider effort as the key to their success. This contrasts with a fixed mindset, where an individual may consider success to be determined by their fixed amount of intelligence.

Further, the extent to which intelligence is believed to be malleable (compared with fixed), and the role of the individual as an active participant in the process, is central to how intelligence mindsets are measured. For example, “You can always greatly change how intelligent you are” (Dweck, 1999, p. 177). In contrast, the Stress Mindset Measure (Crum et al., 2013) is worded such that it conceptualizes stress mindset as fixed‐debilitating on one end of the spectrum and fixed‐enhancing on the other end (debilitating items are reverse‐coded to give an overall index of the extent to which an individual endorses a stress‐is‐enhancing mindset). For example, one item is, “Experiencing stress enhances my performance and productivity” (Crum et al., 2013, p. 732). This fixed conceptualization on both ends of the spectrum contrasts with how stress mindset is described by Crum et al. (2013) and how mindsets are conceptualized more broadly in that the upper end of the spectrum would be expected to encompass beliefs about the malleability of the attribute. In further contrast to how other types of mindset are measured, the wording of the items comprising the Stress Mindset Measure also does not include the role of the individual as an active participant in the process.

The videos used by Crum et al. (2013) to manipulate stress mindset are described as being designed to bias attention toward either the enhancing or debilitating effects of stress, thus eliciting a stress‐is‐enhancing or stress‐is‐debilitating mindset. For example, the stress‐is‐enhancing video presents the view that stress is designed to be enhancing and that one should learn to enjoy and utilize stress. Measuring beliefs about the consequences of stress as fixed‐enhancing and fixed‐debilitating may be appropriate for a manipulation check of attentional bias toward the enhancing or debilitating properties of stress and the Stress Mindset Measure may therefore be appropriate for this purpose. However, it is unclear the extent to which biased attention toward the enhancing effects of stress is responsible for the positive outcomes observed in laboratory studies where these stress mindset manipulations are used, or whether a stable change in mindset is being created. Given that many people can recall examples of when stress was not enhancing in their lives and that Crum et al. (2013) found that people tend to see stress as debilitating by default across their three studies, we anticipate that while attentional bias toward the enhancing consequences of stress may be achieved by these manipulations and measurable by the Stress Mindset Measure, any stable changes in beliefs about stress are likely to reflect a nuanced view of stress encompassing the belief that stress can be both enhancing and debilitating. This potential nuanced composition of beliefs about stress such that they can be both enhancing and debilitating is consequently an important consideration for the measurement of the stress mindset construct.

We therefore contend that measurement of stress mindset should be framed to measure beliefs about the consequences of stress such that stress can be enhancing and that individuals can use stress to be enhancing, on the basis of three arguments that have been detailed above: (i) measuring stress mindset as fixed‐enhancing and fixed‐debilitating contrasts with current theoretical conceptualizations of stress mindset and with (ii) theory on mindsets regarding self‐attributes more broadly; and, (iii) the Stress Mindset Measure may measure just attentional bias toward enhancing or debilitating consequences of stress rather than the extent to which an individual holds balanced beliefs about stress. Therefore, the development and validation of a such a measure will facilitate future research seeking to create enduring changes in stress mindset and to study the potential effects of stress mindset on health and performance outcomes in ecologically valid settings.

### 1.2 The stress control mindset measure

In the current study, we designed a self‐report measure of stress mindset that operationalizes stress mindset as the extent to which an individual holds the belief that the consequences of stress can be enhancing and that the individual can use stress to experience these enhancing consequences. The items were based on the domains of stress mindset conceptualized by Crum et al. (2013) and used in the Stress Mindset Measure: performance and productivity, learning and growth, health and vitality, and a general domain. Consistent with theory regarding mindsets about self‐attributes, the items were also framed to reflect beliefs about malleability of the consequences of the stress response, and to emphasize the role of the individual as an active participant in the process (e.g., “You can use stress to boost your performance and productivity”). The measure contains 15 items and will be referred to as the Stress Control Mindset Measure (SCMM), with the word “control” emphasizing the role of the individual in utilizing the potential positive consequences of stress.

**Figure 1:**
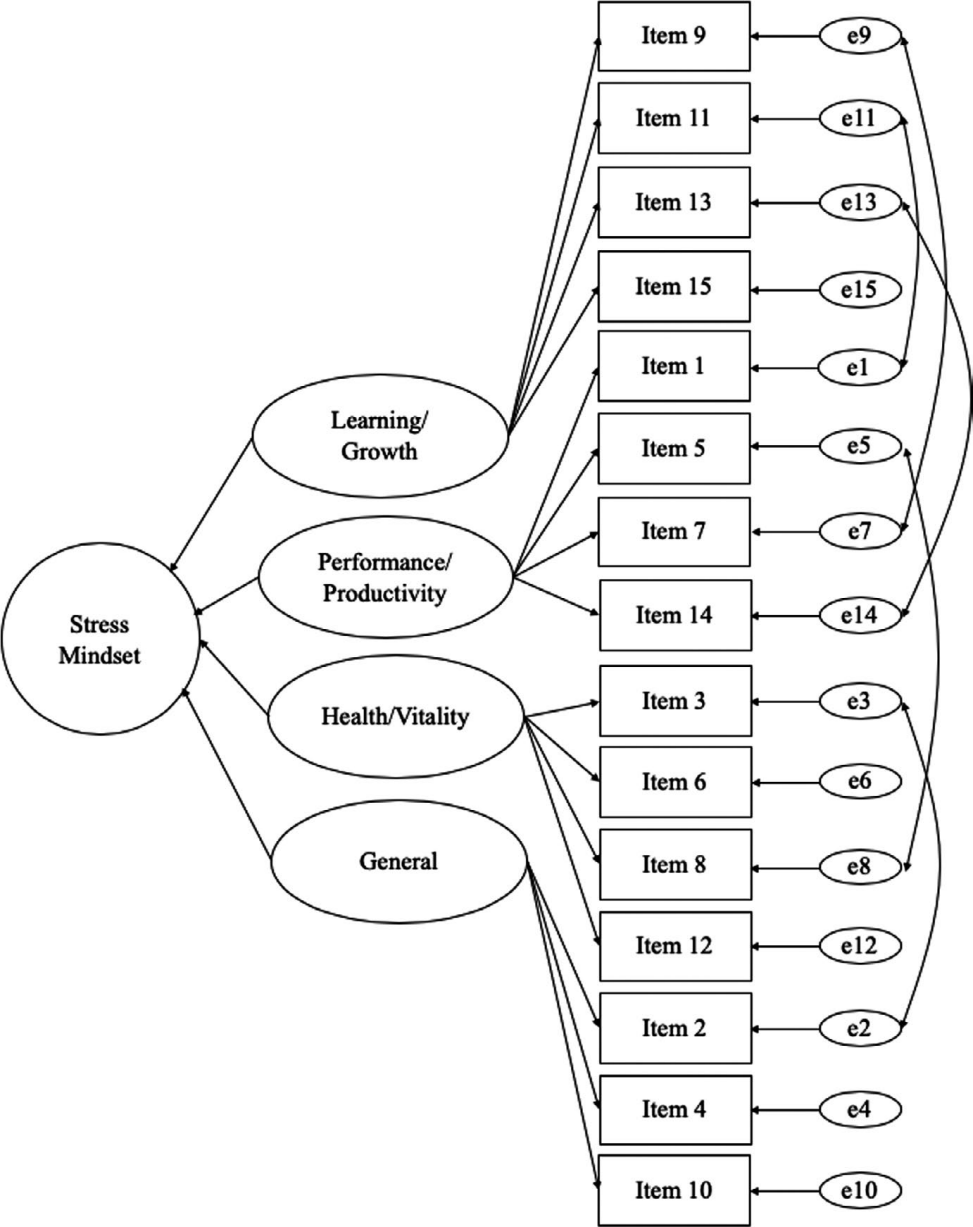
Hypothesized factor structure of the stress control mindset measure. Correlations between error terms (Sample 1; Sample 2): 2 & 3 (0.34; 0.26), 5 & 8 (−0.46; −0.06), 7 & 9 (0.38; 0.04), 1 & 11 (0.39; 0.17), 13 & 14 (0.26; 0.39), General & Health factors (0.66; 0.90)

The study had six aims to determine the psychometric properties of the SCMM in two samples of undergraduate students. We first aimed to refine the items that would comprise the SCMM and to confirm the factor structure. Based on the domains that the measure has been designed around which were consistent with domains in the original Stress Mindset Measure, we expected that the SCMM would form a four‐factor structure, which underpins an overarching second‐order factor: stress mindset. The hypothesized factor structure of the SCMM is presented in Figure 1. It was also expected that the factor structure, factor loadings, intercepts, item error variances, and factor disturbances would be invariant across samples, demonstrating configural, metric, scalar, and strict measurement invariance. Second, we sought to evaluate the reliability of the SCMM using coefficient α, composite reliability, and McDonald's ˥. Our third goal was to examine the convergent validity of the SCMM in relation to existing measures of stress mindset, the Stress Mindset Measure (General and Specific Versions; Crum et al., 2013). Fourth, because stress mindset is theorized as being a distinct construct from appraisals and stressor exposure, we tested the discriminant validity of the SCMM from trait challenge and threat appraisal, amount of stress, and stressor severity appraisal. Fifth, we examined the concurrent validity of the SCMM. Specifically, it was hypothesized that the SCMM would predict psychological well‐being, physical well‐being, perceived stress, proactive behavior under stress, and perceived somatic symptoms. Finally, we examined the incremental validity of the SCMM compared with the Stress Mindset Measure‐General (SMM‐G; Crum et al., 2013). Specifically, it was hypothesized that due to measuring stress mindset in a manner that is consistent with theory regarding how mindsets about self‐attributes operate, the SCMM would explain significantly more variance in psychological well‐being, physical well‐being, perceived stress, proactive behavior under stress, and perceived somatic symptoms than the SMM‐G.

## 2 METHOD

### 2.1 Participants

#### 2.1.1 Sample 1

Participants (*N* = 218; 144 female) were undergraduate university students aged 17–25 years (*M* = 19.26, *SD* = 2.19) recruited from a major university in South‐East Queensland, Australia. Participants were recruited using email and Facebook notices, face‐to‐face at the university, and using posters displayed in common areas at the university. A large majority (75%) of participants indicated their ethnic identity is Australian and 79% of participants were born in Australia. As an incentive for participation, first‐year psychology students were offered course credit, and other participants were offered a voucher for one free coffee and entry into a prize draw for the chance to win a department store gift card valued at AU$50. Sample 1 is part of a data set of a larger project.

#### 2.1.2 Sample 2

Participants (*N* = 214; 141 female) were undergraduate university students aged 18–25 years (*M* = 20.82, *SD* = 1.88) recruited from a major university in the UK. Participants were recruited using email and Facebook notices, face‐to‐face at the university, and using posters displayed in common areas at the university. Just over one‐third (38%) of participants indicated their nationality is British and 35% of participants were born in the UK. The remainder of the sample indicated a diverse range of nationalities and countries of birth. These included countries in Asia, the Middle East, Europe, Africa, and Central and North America. As an incentive for participation, participants were offered entry into a prize draw for the chance to win an Amazon gift card valued at GB£50.

### 2.2 Measures

See online Supplementary Material for full details of all measures used in the study.

#### 2.2.1 Stress control mindset measure (SCMM) scale development

An initial pool of 16 items was constructed to measure the four domains of stress mindset identified by Crum et al. (2013): performance and productivity, learning and growth, health and vitality, and a general domain. In contrast with the Stress Mindset Measure (Crum et al., 2013), the items were designed to reflect malleability of the stress response and the respondent as an active participant in this process (i.e., you can use stress to…) or a fixed stress response (i.e., you are unable to use stress to…). Next, three health psychology experts evaluated the items for construct validity and clarity of wording. As a result, one item was dropped, and several items were modified prior to data collection. This resulted in a final item pool of 15 items (see Table 1). Responses were scored on six‐point Likert scales (1 = *strongly disagree* and 6 = *strongly agree*). Items 1, 4, 6, 10, 11, 12, 13, and 14 are negatively worded.

**Table 1:** Standardized factor loadings in confirmatory factor analyses of SCMM items in samples 1 and 2

	Sample 1	Sample 2
First‐order factors		
Performance and productivity		
1. You are unable to use stress to enhance your performance and productivity (R)	0.612	0.600
5. You can use stress to boost your performance and productivity	0.875	0.865
7. Stress can be used to enhance your performance and productivity	0.875	0.836
14. Stress will impair your performance and productivity (R)	0.655	0.616
Learning and Growth		
9. Stress can be used to enhance your learning and growth	0.777	0.869
11. You are unable to use stress to enhance your learning and growth (R)	0.740	0.740
13. Stress will impair your learning and growth (R)	0.658	0.619
15. You can use stress to facilitate your learning and growth	0.786	0.789
Health and vitality		
3. Stress can be used to enhance your health and vitality	0.736	0.747
6. Stress will impair your health and vitality (R)	0.454	0.307
8. You can use stress to stimulate your health and vitality	0.835	0.905
12. You are unable to use stress to enhance your health and vitality (R)	0.663	0.534
General		
2. Stress can be used as a way to get the most out of your life	0.708	0.664
4. Stress must be reduced or avoided to get the most out of life (R)	0.559	0.411
10. The effect of stress on you is negative (R)	0.644	0.574
Second‐order factor		
Stress mindset		
Performance and productivity	0.929	0.841
Learning and growth	0.942	0.989
Health and vitality	0.644	0.427
General	0.897	0.926

Note

*p* < .001 for all estimates. R = Item is reverse scored.

#### 2.2.2 Stress mindset measure–general (SMM‐G)

The SMM‐G (Crum et al., 2013) is an eight‐item scale measuring stress mindset as the extent to which the belief that stress‐is‐enhancing is endorsed. Participants were asked to indicate on a five‐point Likert scale (0 = *strongly disagree* and 4 = *strongly agree*) the extent to which they agree with each of the statements (e.g., “Experiencing stress enhances my performance and productivity”). The SMM‐G exhibited good internal consistency in both samples (Sample 1, α = 0.81; Sample 2, α = 0.74).

#### 2.2.3 Stress mindset measure–specific (SMM‐S)

The SMM‐S (Crum et al., 2013) is identical to the SMM‐G, except that it asks participants to respond to questions in relation to the primary source of stress in their life currently. The SMM‐S exhibited good internal consistency in both samples (Sample 1, α = 0.83; Sample 2, α = 0.82).

#### 2.2.4 Perceived stress

The Perceived Stress Scale‐10 (PSS‐10) was used to measure perceived stress (Cohen & Williamson, 1988). Participants responded to questions on five‐point Likert scales (0 = *never* and 4 = *very often*). For example, “In the last month, how often have you felt nervous and ‘stressed’?” The PSS‐10 exhibited good internal consistency in both samples (Sample 1, α = 0.88; Sample 2, α = 0.81). The PSS‐10 was scored by reverse scoring negatively worded items and computing a sum of participants’ scores on all items.

#### 2.2.5 Psychological well‐being

The 14‐item Warwick‐Edinburgh Mental Well‐being scale (WEMWBS) was used to measure psychological well‐being (Tennant et al., 2007). The WEMWBS is measured on five‐point Likert scales (1 = *none of the time* to 5 = *all of the time*). For example, “I’ve been feeling good about myself”. The WEMWBS exhibited good internal consistency in both samples (Sample 1, α = 0.92; Sample 2, α = 0.90).

#### 2.2.6 Amount of stress

A single item was used to measure amount of stress. The item was measured on a seven‐point Likert scale (1 = *no stress* and 7 = *an extreme amount of stress*). Participants were asked, “Overall, how much stress do you have in your life right now?” This single‐item measure was used by Crum et al. (2013) and exhibited the same strength of correlation with the Stress Mindset Measure as the social readjustment rating scale (Holmes & Rahe, 1967).

#### 2.2.7 Stressor severity appraisal

A single item was used to measure stressor severity appraisal. The item was measured on a seven‐point Likert scale (1 = *not at all stressful* and 7 = *an extreme amount of stress*). Participants were first asked to indicate the primary source of stress in their life at the moment and were then asked to indicate how stressful they perceive that stressor to be. This measure was also used by Crum et al. (2013) to examine discriminant validity of the Stress Mindset Measure.

#### 2.2.8 Perceived general somatic symptoms

Perceived general somatic symptoms were measured using the 11‐item State‐Trait Inventory for Cognitive and Somatic Anxiety‐Trait (STICSA‐T) somatic subscale (Ree, French, MacLeod, & Locke, 2008; Ree, MacLeod, French, & Locke, 2000). The STICSA‐T is measured on four‐point Likert scales (1 = *almost never* and 4 = *almost always*). The STICSA‐T somatic subscale was scored by computing the sum of all item scores and exhibited good internal consistency in both samples (Sample 1, α = 0.85; Sample 2, α = 0.87).

#### 2.2.9 Physical well‐being

Perceived physical well‐being was measured using the U.S. Centers for Disease Control and Prevention (CDC) Health‐Related Quality of Life Healthy Days (HRQOL‐14) measure (Centers for Disease Control & Prevention., 2000). The measure was developed by the CDC as a shorter alternative to the SF‐36, and validity has been established in several studies (Moriarty, Zack, & Kobau, 2003). The measure was designed to include different indicators, as opposed to psychometrically devised subscales, that can be combined in different ways to fit different uses (Moriarty et al., 2003). Consistent with Keech et al. (2018), we used two items that are measured in days. Participants indicated the number of days their physical health was not good or that pain interfered with their daily activities in the past month (e.g., “Now thinking about your physical health, which includes physical illness and injury, for how many days during the past 30 days was your physical health not good?”) The scores were averaged and subtracted from 30, to give the number of “healthy days” experienced in the past month (Moriarty et al., 2003), and internal consistency was acceptable in both samples (Sample 1, α = 0.70; Sample 2, α = 0.74).

#### 2.2.10 Trait academic stressor appraisals

Trait cognitive appraisal style was measured using the 18‐item version of the Cognitive Appraisal Scale (Skinner & Brewer, 2002). The scale is measured on a six‐point Likert scale (1 = *strongly disagree* and 6 = *strongly agree*) and comprises two subscales: an eight‐item subscale measuring challenge appraisal, and a 10‐item subscale measuring threat appraisal. Prior to completing the measure, participants were asked to read the student scenario from Skinner and Brewer’s (2002) Study 1. The student scenario is a vignette that asks participants to imagine themselves as being about to take an exam of an uncertain difficulty that will determine their progression to an important subject in the following year. This administration of the measure is therefore indicative of trait cognitive appraisals of a specific type of stressor. Both the challenge (Sample 1, α = 0.81; Sample 2, α = 0.77) and threat (Sample 1, α = 0.94; Sample 2, α = 0.91) appraisal subscales exhibited good internal consistency.

#### 2.2.11 Proactive coping behavior

Proactive coping behavior was measured using the six‐item Proactive Under Stress Scale (Keech et al., 2018). Participants were asked to indicate on a five‐point Likert scale (1 = *never* and 5 = *very often*) the extent to which they were proactive, engaged in planning, or avoided procrastination while under stress or to cope with stress in the past month. For example, “In the last month, how often did you avoid procrastination to cope with stress?”. Internal consistency was acceptable in Sample 1 (α = 0.77) and Sample 2 (α = 0.61).

### 2.3 Design and procdure

Cross‐sectional designs were used for both Sample 1 and Sample 2. Data collection for each sample was approved by the Griffith University Human Research Ethics Committee for Sample 1 (reference: 2015/723) and the Faculty of Science and Health Ethics Sub‐Committee at the University of Essex for Sample 2 (reference: SO1607). Sample 1 participants completed study measures in a research laboratory in the psychology department between March and October 2016 while Sample 2 participants completed study measures online at a time of their convenience between December 2016 and February 2017. The measures were contained within a survey hosted in an online survey tool (Qualtrics™). To determine target sample sizes for the current study, we followed the rule of thumb developed by Myers, Ahn, and Jin (2011) based on their Monte Carlo simulations for determining sample size in M*plus* (Muthén & Muthén, 2002). Specifically, Myers et al. (2011) recommend *N* ≥ 200 for CFA of a theoretical model. We also computed post hoc statistical power based on RMSEA (MacCallum, Browne, & Sugawara, 1996) using the *WebPower* package in *R* (Zhang & Yuan, 2018). This revealed statistical power estimates of 0.96 for Sample 1 and 1.00 for Sample 2.

#### 2.3.1 Statistical analysis

##### 2.3.1.1 Factorial validity

Four factors of the SCMM were determined a priori to measure stress mindset in the following contexts/domains: performance and productivity, learning and growth, health and vitality, and a general factor. As the four factors were hypothesized to underpin an overarching construct, stress mindset, a second‐order CFA was conducted using M*plus* version 7.4 (Muthén & Muthén, 2015). Specifically, the higher order stress mindset construct is expected to be underpinned by beliefs about stress in the context of performance and productivity, learning and growth, health and vitality, and in general. Maximum‐likelihood estimation with robust standard errors was used due to the presence of minor skewness and multivariate outliers. As this did not produce a substantial change in the overall strength and direction of results compared with maximum‐likelihood estimation, final analyses were conducted using standard maximum‐likelihood estimation. The measurement models were evaluated against conventional standards of model fit (Hu & Bentler, 1999).

##### 2.3.1.2 Measurement invariance

Measurement invariance of the SCMM between the samples from Australia and the UK was tested using multi‐group CFA in M*plus* version 7.4 (Muthén & Muthén, 2015). We used a seven‐step procedure outlined by (Dimitrov, 2017) to test for configural (equal factor structure), metric (equal first‐ and second‐order factor loadings), scalar (equal first‐order factor and indicator intercepts), and item and factor uniqueness (equal item error variances and factor disturbances) strict invariance of the second‐order factor model.

##### 2.3.1.3 Reliability

Several indicators of reliability were calculated. Omega (total) ˥ was calculated according to McNeish’s (2018) guidelines. Composite reliability coefficients were calculated using Colwell’s (2016) composite reliability calculator which applied Raykov’s (1997) method.

##### 2.3.1.4 Convergent validity

The convergent validity of the SCMM was investigated in Samples 1 and 2 by computing bivariate correlations and 95% CIs about the correlations between the SCMM and the SMM‐G and SMM‐S (Crum et al., 2013) using SPSS V25.0.

##### 2.3.1.5 Discriminant validity

The discriminant validity of the SCMM was investigated in both samples by computing bivariate correlations and 95% CIs about the correlation between the SCMM and trait challenge and threat appraisal, amount of stress, and stressor severity appraisal using SPSS V25.0.

##### 2.3.1.6 Concurrent validity

A series of linear multiple regression analyses were conducted using SPSS V25.0 to examine concurrent validity of the SCMM in cross‐sectionally predicting proactive behavior, perceived somatic symptoms, psychological well‐being, perceived stress, and physical well‐being in both samples.

##### 2.3.1.7 Incremental validity

A series of stepwise linear multiple regressions using SPSS V25.0 were conducted to investigate whether the SCMM explains unique variance over and above that explained by the SMM‐G (Crum et al., 2013). For each analysis, the SMM‐G was entered into the equation in Step 1, and the SCMM was entered in Step 2.

## 3 **RESULTS** 1

### 3.1 Preliminary analyses: sample 1 and sample 2

Normality for each of the SCMM items was assessed based on ratio of skew to *SE *> ±3.29 and ratio of kurtosis to *SE* kurtosis > ±3.29. Minor univariate skewness was indicated on six items in Sample 1 and four items for Sample 2. Univariate kurtosis was not indicated in either sample. Standardized *z* scores for each variable were saved to evaluate univariate outliers. An outlier was indicated when *z *> ±3.29. No univariate outliers were detected in either sample. Mahalanobis distance indicated the presence of five multivariate outliers in Sample 1 and six multivariate outliers in Sample 2. There were no missing data. Negatively worded items were reverse‐coded for all analyses.

### 3.2 Factorial validity

Initial analysis of the hypothesized structure with the Sample 1 data yielded poor model fit (χ^2^ (86) = 422.61, *p* < .001, CFI = 0.827, TLI = 0.789, SRMR = 0.067, RMSEA = 0.134). Further investigation of the shared error variance between items revealed the presence of a measurement artefact attributable to the reverse‐coded negatively worded items, which according to Brown (2014) and Motl and DiStefano (2002) can be a cause of model misfit. Therefore, a latent factor was defined to account for the shared method variance between reverse‐coded items (Motl & DiStefano, 2002). Specifically, all of the negatively worded items were set to indicate a latent method factor. Because the default assumption of uncorrelated residuals may be a source of misfit for similarly worded items (Brown, 2014), residuals were allowed to covary for similarly worded items (Items: 2 & 3, 5 & 8, 7 & 9, 1 & 11, 13 & 14). Due to conceptual similarities between experiencing health and vitality and the “getting the most from life” wording from the general domain, the general and health/vitality first‐order factors were also allowed to correlate (items are presented in Table 1). The final second‐order CFA model indicated adequate model fit and a consistent four‐factor structure in Sample 1 from Australia (χ^2^ (72) = 122.25, *p* < .001, CFI = 0.974, TLI = 0.962, SRMR = 0.036, RMSEA = 0.057), and this was replicated in Sample 2 from the UK (χ^2^ (72) = 158.35, *p* < .001, CFI = 0.949, TLI = 0.925, SRMR = 0.043, RMSEA = 0.075). Item wording and factor loadings from both samples are presented in Table 1. Inter‐item correlations, and item means and standard deviations are presented in online Supplemental Material for both samples.

### 3.3 Measurement invariance

Full measurement invariance was supported when the model fit of the metric, scalar, and uniqueness invariance models did not differ substantially from the configural model as indicated by ΔCFI < 0.01 (Cheung & Rensvold, 2002). Results of invariance testing analyses are presented in Table 2. All models exhibited a good fit with the data, with ΔCFI indicating no substantial differences in model fit between the configural, metric, scalar, and strict models. We therefore concluded that SCMM exhibited good measurement invariance across samples, indicating equality in factor structure, first‐ and second‐order factor loadings, first‐order factor and indicator intercepts, and item error variances and factor disturbances.

**Table 2:** Model fit and measurement invariance tests across samples

	χ^2^	*df*	χ^2^_diff_	Δdf	*p*	RMSEA (90% CI)	CFit	SRMR	CFI	ΔCFI	TLI
Single‐group solutions											
Sample 1 (AU) (*n* = 218)	122.25***	72	‐	‐	‐	0.057		0.036	0.974	‐	0.962
Sample 2 (UK) (*n* = 214)	158.35***	72	‐	‐	‐	0.075		0.043	0.949	‐	0.925
Measurement invariance											
Equal form (factor structures)	280.60***	144	‐	‐	‐	0.066	0.012	0.040	0.962	‐	0.945
Equal first‐order factor loadings	299.83***	162	19.23	18	0.378	0.063	0.031	0.052	0.962	−0.000	0.951
Equal second‐order factor loadings	310.25***	165	29.65	21	0.099	0.064	0.021	0.066	0.960	−0.002	0.949
Equal indicator intercepts	333.19***	179	52.59	35	0.028	0.063	0.022	0.068	0.958	−0.004	0.950
Equal first‐order factor intercepts	334.30***	180	53.70	36	0.029	0.063	0.023	0.068	0.958	−0.004	0.950
Equal first‐order factor disturbances	343.27***	185	62.67	41	0.016	0.063	0.022	0.064	0.956	−0.006	0.951
Equal item residual variances	352.23***	189	71.63	45	0.007	0.063	0.019	0.065	0.955	−0.007	0.950

*** *p* < .001

### 3.4 Reliability

The complete scale exhibited excellent internal consistency and the four subscales exhibited good internal consistency in both samples. The composite reliabilities (ρ), coefficient α and McDonald's ˥ for the complete scale, the subscales, and the second‐order factors indicated adequate reliability (see Table 3). Test–retest reliability was evaluated across three time‐points using data (*n* = 70) from the control group in Keech, Hagger, and Hamilton (2019), where Time 1 was measured at the start of a 40‐min laboratory session, Time 2 was measured at the end of that session, and Time 3 was measured two‐weeks later. In this laboratory session, participants completed a battery of measures and completed an inert mental imagery exercise where they were instructed to imagine cutting and smelling a lemon (see Keech et al., 2019. for full list of measures and details of the imagery task). Correlations between administrations of the SCMM indicated good test–retest reliability: Time 1 to Time 2, *r* = 0.91, *p* < .001; Time 2 to Time 3 *r* = 0.92, *p* < .001; Time 1 to Time 3, *r* = 0.88, *p* < .001.

**Table 3:** Reliability statistics for SCMM in sample 1 and sample 2

	Sample 1			Sample 2		
	ρ	˥	α	ρ	˥	α
Single‐factor	0.938	0.949	0.926	0.929	0.941	0.902
Second‐order factor	0.919	0.919	0.885	0.888	0.888	0.842
Performance and productivity subscale	0.845	0.882	0.853	0.824	0.851	0.828
Learning and growth subscale	0.830	0.864	0.851	0.843	0.876	0.862
Health and vitality subscale	0.774	0.795	0.783	0.735	0.763	0.701
General subscale	0.673	0.706	0.720	0.569	0.618	0.641

Note

ρ = Composite reliability coefficient; ˥ = Omega (total); α = Coefficient alpha.

### 3.5 Convergent validity

The correlations between the SCMM and the SMM‐G and SMM‐S (Crum et al., 2013) are presented in Table 4. As anticipated, the SCMM exhibited strong correlations with the SMM‐G and SMM‐S in both samples, supporting convergent validity of the SCMM with the existing measure of stress mindset. The four SCMM subscales also exhibited strong correlations with the SMM‐G and moderate–strong correlations with the SMM‐S in both samples.

**Table 4:** Correlations and 95% confidence intervals between the stress control mindset measure (SCMM) and its subscales, and other conceptually related measures, and means and standard deviations for the SCMM and SCMM subscales

Variable	SCMM	Performance and productivity subscale	Health and vitality subscale	Learning and growth subscale	General subscale	*M (SD)*
SMM‐G^a^ ‐ S1^d^	0.79*** [0.73, 0.84]	0.69*** [0.61, 0.76]	0.66*** [0.57, 0.73]	0.67*** [0.58, 0.74]	0.72*** [0.65, 0.79]	2.74 (0.61)
‐ S2^e^	0.73*** [0.65, 0.80]	0.62*** [0.53, 0.71]	0.49*** [0.36, 0.59]	0.65*** [0.55, 0.72]	0.64*** [0.54, 0.71]	1.69 (0.60)
SMM‐S^b^ ‐ S1	0.64*** [0.54, 0.73]	0.52*** [0.41, 0.63]	0.53*** [0.42, 0.63]	0.58*** [0.47, 0.67]	0.60*** [0.49, 0.69]	2.64 (0.67)
‐ S2	0.66*** [0.57, 0.74]	0.53*** [0.43, 0.63]	0.49*** [0.37, 59]	0.57*** [0.46, 0.66]	0.59*** [0.49, 0.69]	1.61 (0.71)
Amount of Stress ‐ S1	−0.20** [−0.33, −0.06]	−0.14* [−0.28, 0.00]	−0.20** [−0.33, −0.07]	−0.16* [−0.29, −0.02]	−0.19** [−0.33, −0.04]	4.52 (1.15)
‐ S2	−0.17* [−0.29, −0.04]	−0.06 [−0.19, 0.07]	−0.20*** [−0.34, −0.06]	−0.15* [−0.27, −0.02]	−0.16* [−0.29, −0.03]	4.37 (1.43)
Stressor Severity Appraisal ‐ S1	−0.24*** [−0.36, −0.11]	−0.19** [−0.32, −0.07]	−0.25*** [−0.38, −0.11]	−0.18** [−0.31, −0.05]	−0.19** [−0.32, −0.06]	5.45 (1.23)
‐ S2	−0.20** [−0.32, −0.08]	−0.08 [−0.21, 0.06]	−0.28*** [−0.42, −0.14]	−0.17* [−0.30, −0.04]	−0.15* [−0.28, −0.02]	5.24 (1.48)
Challenge Appraisal^c^ ‐ S1	0.28*** [0.16, 0.39]	0.30*** [0.18, 0.42]	0.20** [0.07, 0.32]	0.27*** [0.15, 0.38]	0.17* [0.04, 0.30]	32.86 (6.40)
‐ S2	0.26*** [0.11, 0.39]	0.18** [0.02, 0.34]	0.21** [0.05, 0.34]	0.22** [0.08, 0.36]	0.23** [0.09, 0.36]	33.24 (6.35)
Threat Appraisal^c^ ‐ S1	−0.24*** [−0.37, −0.09]	−22** [−0.36, −0.08]	−0.10 [−0.24, 0.04]	−0.29*** [−0.41, −0.16]	−0.18* [−0.32, −0.04]	39.18 (12.15)
‐ S2	−0.37*** [−0.49, −0.24]	−0.26*** [−0.39, −0.11]	−0.32*** [−0.44, −0.19]	−0.34*** [−0.46, −0.21]	−0.30*** [−0.42, −0.17]	40.21 (10.97)
*M (SD)* ‐ S1‐S2	3.28 (0.81)	3.77 (0.97)	2.63 (0.86)	3.65 (0.96)	3.02 (0.96)	‐
	3.21 (0.80)	3.72 (0.99)	2.51 (0.86)	3.49 (1.07)	3.07 (0.98)	‐

^a^ SMM‐G = Stress mindset measure—general (Crum et al., 2013).

^b^ SMM‐S = Stress mindset measure—specific (Crum et al., 2013).

^c^ Cognitive Appraisal Scale (Skinner & Brewer, 2002).

^d^ S1—Sample 1.

^e^ S2 = Sample 2.

* *p* < .05.

** *p* < .01.

*** *p* < .001.

### 3.6 Discriminant validity

As anticipated, the correlations between the SCMM and challenge and threat appraisal, amount of stress, and stressor severity appraisal were relatively small (*r*s < |0.38|), indicating distinctiveness between the constructs. Further, 95% CIs about the correlations between the overall SCMM and trait challenge and threat appraisal, amount of stress, and stressor severity appraisal ranged from |0.04| to |0.49|. We also examined the correlations and 95% CIs about the correlations between the SCMM subscales and trait challenge and threat appraisal, amount of stress, and stressor severity appraisal. As anticipated, the correlations were relatively small (*r*s < |0.34|), with 95% CIs ranging from 0.00 to |0.46|, indicating distinctiveness between the constructs. Taken together, this provides support for discriminant validity. See Table 4 for correlations and 95% CIs.

### 3.7 Concurrent validity

In Sample 1, the SCMM significantly predicted proactive behavior (β = 0.27, *R* ^2^ = 0.07, *p* < .001), perceived somatic symptoms (β = −0.24, *R* ^2^ = 0.06, *p* < .001), psychological well‐being (β = 0.27, *R* ^2^ = 0.07, *p* < .001), perceived stress (β = −0.38, *R* ^2^ = 0.14, *p* < .001), and physical well‐being (β = 0.26, *R* ^2^ = 0.07, *p* < .001). In Sample 2, the SCMM significantly predicted proactive behavior (β = 0.19, *R* ^2^ = 0.04, *p* = .007), psychological well‐being (β = 0.32, *R* ^2^ = 0.10, *p* < .001), perceived stress (β = −0.35, *R* ^2^ = 0.12, *p* < .001), and physical well‐being (β = 0.25, *R* ^2^ = 0.06, *p* < .001); however, the SCMM did not significantly predict perceived somatic symptoms (β = −0.11, *R* ^2^ = 0.01, *p* = .109). See Table 5 for regression results for tests of concurrent validity.

**Table 5:** Stepwise regressions of stress mindset measure‐general (SMM‐G; Crum et al., 2013) and stress control mindset measure (SCMM) predicting stress‐related Outcomes in five models examining incremental validity

Dependent variable		Proactive behavior^c^	Somatic symptoms^d^	Psychological well‐being^e^	Perceived stress^f^	Physical Well‐being^g^
Sample 1						
Step 1: SMM‐G^a^	β	0.217**	−0.187**	0.206**	−0.351***	0.207**
	*R* ^2^	0.047**	0.035**	0.042**	0.123***	0.043**
Step 2: SMM‐G^a^	β	0.011	0.004	−0.011	−0.141	0.004
SCMM^b^	β	0.261*	−0.242*	0.275*	−0.266*	0.256*
	*R* ^2^	0.073***	0.057**	0.071***	0.150***	0.067**
	Δ*R* ^2^	0.025*	0.022*	0.028*	0.026*	0.059*
*M (SD)*		2.93 (0.66)	19.63 (6.09)	44.45 (9.44)	21.91 (6.87)	25.28 (5.49)
Sample 2						
Step 1: SMM‐G^a^	β	0.189*	−0.226**	0.273***	−0.297***	0.216**
	*R* ^2^	0.032*	0.051**	0.075***	0.088***	0.047**
Step 2: SMM‐G^a^	β	0.088	−0.033	0.079	−0.091	0.075
SCMM^b^	β	0.122	−0.262*	263**	−0.279**	0.191
	*R* ^2^	0.039*	0.073***	0.106***	0.124***	0.063**
	Δ*R* ^2^	0.007	0.031*	0.032**	0.036**	0.017
*M (SD)*		2.96 (0.63)	19.15 (6.16)	44.77 (9.22)	22.47 (6.75)	24.03 (5.42)

^a^ Stress mindset measure—general (SMM‐G; Crum et al., 2013).

^b^ Stress control mindset measure (SCMM).

^c^ Proactive under stress scale (Keech et al., 2018).

^d^ STICSA‐T Somatic subscale (Ree et al., 2008).

^e^ Warwick‐Edinburgh Mental Well‐being Scale (WEMWBS; Tennant et al., 2007).

^f^ Perceived stress scale (PSS‐10; Cohen & Williamson, 1988).

^g^ CDC Healthy Days.

* *p* < .05.

** *p* < .01.

*** *p* < .001.

### 3.8 Incremental validity

An examination of change in *R* ^2^ across the analyses indicated that the SCMM explained significantly more variance than the SMM‐G in all of the measured constructs with additional variance explained ranging from 2.2% to 5.9% in Sample 1, providing support for incremental validity. These results were partially replicated in Sample 2, with the SCMM explaining between 3.1% and 3.6% more variance in perceived somatic symptoms, psychological well‐being, and perceived stress than the SMM‐G. These results, however, were not replicated for proactive behavior or physical well‐being, with a non‐significant increase in *R* ^2^. See Table 5 for results specific to each variable.

## 4 DISCUSSION

The current study reported the development and assessment of the psychometric properties of the SCMM in two samples from Australia and the UK. The SCMM was developed to measure stress mindset as the extent to which an individual holds the belief that the consequences of stress can be enhancing and that the individual can use stress to be enhancing. The results of the CFAs on Sample 1 provided initial evidence for the factorial validity of the SCMM in a sample of Australian undergraduate university students. The results of the analyses on Sample 2 corroborated the factorial validity of the SCMM in an ethnically diverse sample of British undergraduate university students. A series of multi‐group CFAs also indicated that the SCMM demonstrated invariant structure, factor loadings, intercepts, and item and factor uniqueness across samples. Additionally, the findings demonstrate the convergent and discriminant validity of the SCMM. Specifically, the overall SCMM and all subscales exhibit a moderate to strong relationship with the original measures of stress mindset (SMM‐G, SMM‐S), supporting convergent validity of the SCMM. It was also demonstrated across samples that the overall SCMM and all subscales are distinct from trait challenge and threat appraisals, the amount of stress participants were experiencing, and the perceived severity of the primary source of stress in their life, providing support for discriminant validity.

Concurrent validity of the SCMM in predicting proactive behavior, psychological well‐being, perceived stress, and physical well‐being was also supported in both samples. Concurrent validity of the SCMM in predicting perceived somatic symptoms was supported in Sample 1 but not Sample 2. We also assessed the incremental validity of the SCMM against the SMM‐G in both samples. In Sample 1, the SCMM explained significantly more variance than the SMM‐G in all of the measured constructs, providing support for incremental validity. These results were partially replicated in Sample 2, with the SCMM explaining significantly more variance in perceived somatic symptoms, psychological well‐being, and perceived stress than the SMM‐G. These results, however, were not replicated for proactive behavior or physical well‐being, with a non‐significant increase in variance explained. It is noteworthy that there was a substantial difference in internal consistency of the measure of proactive behavior between samples with the scale in Sample 2 exhibiting questionable internal consistency. It is possible that this is responsible for the discrepancy in results. Further, physical well‐being was measured using two self‐report items where participants retrospectively provided their perception of their physical well‐being in the past month. It is possible that a non‐self‐report measure of health or the use of ecological momentary assessment methods would provide a more accurate measure for this kind of assessment. This would allow a stronger conclusion to be drawn about the relationship between stress mindset and physical health.

### 4.1 Theoretical and practical implications

The current findings indicate that the SCMM is a reliable and valid measure that cross‐sectionally predicts proactive behavior, perceived somatic symptoms, psychological well‐being, perceived stress, and physical well‐being. Future studies can therefore apply this measure when seeking to further understand how stress mindset may influence outcomes under stress. A further practical implication is that the SCMM may be used to evaluate interventions aiming to encourage more adaptive responses to stress because it conceptualizes and measures stress mindset as the extent to which an individual believes that stress can be enhancing–a balanced view of stress. Specifically, stress mindset interventions in the future may seek to present a balanced view of stress or that stress can be enhancing. This is supported by the evidence for incremental validity attained in the current study, and is consistent with Liu, Vickers, Reed, and Hadad (2017), who found that presenting videos outlining balanced consequences of stress results in significantly lower heart rates and diastolic blood pressure following a laboratory‐induced stressor than when videos outlining strictly positive or negative consequences of stress are presented.

The finding that the SCMM demonstrated incremental validity, explaining significantly more variance (between 2.2% and 5.9%) than the SMM‐G in all outcomes in Sample 1 and all but two outcomes in Sample 2, also has both practical and theoretical implications. The practical implication of these findings is that researchers can use the SCMM to measure stress mindset to improve measurement precision and minimize measurement error. This is important because estimates of the associations between constructs are closer to true population values when error in measurement is kept to a minimum. In terms of theoretical implications, the incremental validity provides preliminary evidence that conceptualizing stress mindset as a spectrum of beliefs ranging from the consequences of stress being debilitating to the belief that stress can be enhancing may be more effective in explaining stress‐related outcomes than measuring stress mindset as fixed‐debilitating to fixed‐enhancing. A further theoretical implication is that the stress is debilitating items load on the same factors as the stress can be enhancing items. This is consistent with what has been observed with the SMM‐G (Crum et al., 2013) and supports that beliefs that stress can be enhancing and stress is debilitating can be conceptualized as a spectrum of beliefs rather than two independent beliefs operating in parallel.

### 4.2 Strengths, limitations, and future directions

In the current study, we sought to develop and evaluate the psychometric properties of a theoretically consistent measure of stress mindset. A particular strength of the study is that the findings have been tested in two samples from different countries. The Australian sample was relatively ethnically homogeneous, while the British sample was more ethnically diverse. Data for the Australian sample were also collected in a research laboratory, while data for the British sample were collected online. This allowed us to determine that the factor structure of the measure held across a range of contexts. To the authors’ knowledge, this is the first study to evaluate the psychometric properties and measurement invariance of a measure of stress mindset across two international samples. The study must, however, be viewed in light of some limitations. Namely, the data were collected cross‐sectionally for both samples, meaning that we were unable to determine temporal precedence among constructs, establishing concurrent rather than predictive validity. Similarly, longitudinal measurement invariance could not be tested using this design. It is noteworthy that the SCMM showed only a weak correlation with the amount of stress participants were experiencing and that the SCMM exhibited high test–retest reliability. We therefore expect that the SCMM is likely to produce stable and invariant measurement over time while stress levels fluctuate but suggest that future research should seek to longitudinally examine within‐person variation in stress mindset using the SCMM.

A further limitation is that the current study relied on retrospective recall of responses to stress which prevents external validity of the measure from being assessed. Future research should therefore seek to examine whether the SCMM can predict non‐self report measures of subsequent behavioral responses to stress. Another important future direction is to understand how the domains of stress mindset that are represented by the SCMM subscales may be affected by different types of stressful life events, and whether they differentially influence stress‐related health and performance outcomes. A strength of the SCMM is that it contains four subscales pertaining to these domains, which can be used in future investigation of these domains. It is also possible that these domains, which were developed by Crum et al. (2013) using focus groups with research laboratory members, do not cover all beliefs about the consequences of stress. For example, a recent qualitative study has identified that stress beliefs may also comprise domains such as beliefs about the consequences of stress on cognition, interpersonal factors, confidence, and emotion (Kilby, Sherman, & Wuthrich, 2020). Future research could seek to measure these additional domains to examine whether they explain additional variance in stress‐related outcomes above and beyond that explained by measurement of the domains already utilized in the SCMM.

While the current study sought to measure beliefs about stress in a way that is characteristic of a mindset (i.e., a set of beliefs with a certain structure, characteristics, and downstream consequences), it was beyond the scope of the current study to examine whether this is a better approach than simply looking at explicit beliefs about stress. Future research should seek to examine stress mindset alongside measures that have emerged as recent developments in the stress beliefs literature such as the Beliefs About Stress Scale (Laferton, Stenzel, & Fischer, 2018).

### 4.3 CONCLUSION

The current study demonstrated that the SCMM is a valid and reliable measure of stress mindset with good psychometric properties. Preliminary evidence for the reliability and validity of the SCMM was obtained through analysis of Sample 1 data from Australia, and these findings were corroborated through analysis of Sample 2 data from the UK. Specifically, the SCMM measures stress mindset as the extent to which individuals believe that the consequences of stress can be enhancing, and that they can use stress to be enhancing. The measure is theoretically consistent, is related to key stress‐related outcomes, and should be considered as a useful measure of stress mindset for future research aimed at understanding, changing, and tracking stress mindsets across time.

## CONFLICT OF INTEREST

The authors declare that they have no conflicts of interest.

## AUTHOR CONTRIBUTIONS

Study conceptualization and development: Jacob J. Keech, Kyra Hamilton, Martin S. Hagger, Sheina Orbell, and Frances V. O’Callaghan; Data collection: Jacob J. Keech, Sheina Orbell; Data curation and analysis: Jacob J. Keech; Writing—original draft: Jacob J. Keech, Kyra Hamilton, Martin S. Hagger, Sheina Orbell, and Frances V. O’Callaghan; Writing—review and editing: Jacob J. Keech, Kyra Hamilton, Martin S. Hagger, Sheina Orbell, and Frances V. O’Callaghan.

### Peer Review

The peer review history for this article is available at https://publons.com/publon/10.1002/brb3.1963.

## Supporting information

Supinfo

## Data Availability

The data file, analysis code, and output files from all analyses can be accessed on the project website at: https://osf.io/6kdgz/

## Footnotes

^1^ The dataset, analysis scripts, and analysis outputs are available from https://osf.io/6kdgz
