# Supplementary material for: Psychometric properties of the stress control mindset measure in university students from Australia and the UK
Source: Brain Behav. 2020 Nov 24;11(2):e01963. doi: 10.1002/brb3.1963 (PMC7882182; doi:10.1002/brb3.1963)
Supplement: Supplementary file 1 — Supinfo [file BRB3-11-e01963-s001.docx]

Supplemental Online Material 1

*Inter-item Correlations, Means and Standard Deviations for SCMM Items in Sample 1*

|  | 1 | 2 | 3 | 4 | 5 | 6 | 7 | 8 | 9 | 10 | 11 | 12 | 13 | 14 | 15 | M (SD) |
| --- | --- | --- | --- | --- | --- | --- | --- | --- | --- | --- | --- | --- | --- | --- | --- | --- |
| Item 1* | – |  |  |  |  |  |  |  |  |  |  |  |  |  |  | 3.78 (1.27) |
| Item 2 | .35 | – |  |  |  |  |  |  |  |  |  |  |  |  |  | 3.25 (1.20) |
| Item 3 | .23 | .57 | – |  |  |  |  |  |  |  |  |  |  |  |  | 2.39 (1.08) |
| Item 4* | .41 | .45 | .33 | – |  |  |  |  |  |  |  |  |  |  |  | 3.14 (1.18) |
| Item 5 | .52 | .54 | .35 | .40 | – |  |  |  |  |  |  |  |  |  |  | 4.02 (1.12) |
| Item 6* | .25 | .31 | .38 | .36 | .26 | – |  |  |  |  |  |  |  |  |  | 2.73 (1.05) |
| Item 7 | .57 | .49 | .34 | .41 | .75 | .19 | – |  |  |  |  |  |  |  |  | 3.91 (1.08) |
| Item 8 | .26 | .45 | .61 | .37 | .33 | .39 | .49 | – |  |  |  |  |  |  |  | 2.64 (1.05) |
| Item 9 | .43 | .51 | .37 | .32 | .54 | .24 | .71 | .48 | – |  |  |  |  |  |  | 3.83 (1.13) |
| Item 10* | .49 | .43 | .42 | .50 | .48 | .46 | .44 | .47 | .45 | – |  |  |  |  |  | 2.68 (1.23) |
| Item 11* | .71 | .48 | .37 | .42 | .59 | .31 | .57 | .32 | .56 | .58 | – |  |  |  |  | 3.71 (1.24) |
| Item 12* | .42 | .40 | .55 | .33 | .39 | .37 | .34 | .55 | .29 | .49 | .50 | – |  |  |  | 2.73 (1.22) |
| Item 13* | .55 | .47 | .40 | .39 | .51 | .44 | .48 | .35 | .51 | .62 | .69 | .48 | – |  |  | 3.38 (1.15) |
| Item 14* | .63 | .46 | .36 | .41 | .60 | .41 | .52 | .39 | .44 | .59 | .64 | .53 | .73 | – |  | 3.38 (1.18) |
| Item 15 | .48 | .45 | .34 | .33 | .63 | .18 | .61 | .29 | .63 | .41 | .61 | .34 | .54 | .49 | – | 3.68 (1.08) |

*Note.* All inter-item correlations significant at *p* < .01.

Supplemental Online Material 2

*Inter-item Correlations, Means and Standard Deviations for SCMM Items in Sample 2*

|  | 1 | 2 | 3 | 4 | 5 | 6 | 7 | 8 | 9 | 10 | 11 | 12 | 13 | 14 | 15 | M (SD) |
| --- | --- | --- | --- | --- | --- | --- | --- | --- | --- | --- | --- | --- | --- | --- | --- | --- |
| Item 1* | – |  |  |  |  |  |  |  |  |  |  |  |  |  |  | 3.82 (1.26) |
| Item 2 | .42 | – |  |  |  |  |  |  |  |  |  |  |  |  |  | 3.39 (1.26) |
| Item 3 | .21 | .44 | – |  |  |  |  |  |  |  |  |  |  |  |  | 2.33 (1.15) |
| Item 4* | .32 | .29 | .21 | – |  |  |  |  |  |  |  |  |  |  |  | 3.10 (1.29) |
| Item 5 | .53 | .49 | .24 | .23 | – |  |  |  |  |  |  |  |  |  |  | 3.90 (1.19) |
| Item 6* | .06^ns^ | .07^ns^ | .19 | .16 | .09^ns^ | – |  |  |  |  |  |  |  |  |  | 2.68 (1.20) |
| Item 7 | .53 | .47 | .26 | .23 | .73 | .06^ns^ | – |  |  |  |  |  |  |  |  | 3.86 (1.19) |
| Item 8 | .20 | .34 | .66 | .34 | .23 | .30 | .19 | – |  |  |  |  |  |  |  | 2.53 (1.16) |
| Item 9 | .41 | .57 | .33 | .29 | .61 | .23 | .57 | .38 | – |  |  |  |  |  |  | 3.71 (1.23) |
| Item 10* | .42 | .37 | .35 | .46 | .38 | .41 | .32 | .38 | .51 | – |  |  |  |  |  | 2.73 (1.29) |
| Item 11* | .56 | .42 | .13^ns^ | .41 | .56 | .23 | .51 | .22 | .64 | .55 | – |  |  |  |  | 3.44 (1.40) |
| Item 12* | .36 | .24 | .35 | .32 | .20 | .24 | .14 | .50 | .20 | .42 | .40 | – |  |  |  | 2.52 (1.26) |
| Item 13* | .44 | .45 | .17 | .44 | .46 | .33 | .43 | .24 | .55 | .56 | .66 | .32 | – |  |  | 3.28 (1.19) |
| Item 14* | .43 | .38 | .08 | .39 | .53 | .34 | .54 | .16 | .42 | .51 | .62 | 29 | .73 | – |  | 3.29 (1.24) |
| Item 15 | .43 | .54 | .30 | .27 | .58 | .14 | .51 | .31 | .68 | .39 | .64 | .27 | .49 | .51 | – | 3.53 (1.26) |

*Note*. All inter-item correlations significant at *p* < .01 unless otherwise specified. ^ns^non-significant

Supplemental Online Material 3. *Items and Response Scales for Study Variables*

| Construct | Measure | Items | Scale/Scoring |
| --- | --- | --- | --- |
| Stress mindset | SCMM (Keech et al., 2018) | You are unable to use stress to enhance your performance and productivity*  Stress can be used as a way to get the most out of your life  Stress can be used to enhance your health and vitality  Stress must be reduced or avoided to get the most out of life*  You can use stress to boost your performance and productivity  Stress will impair your health and vitality*  Stress can be used to enhance your performance and productivity  You can use stress to stimulate your health and vitality  Stress can be used to enhance your learning and growth  The effect of stress on you is negative*  You are unable to use stress to enhance your learning and growth*  You are unable to use stress to enhance your health and vitality*  Stress will impair your learning and growth*  Stress will impair your performance and productivity*  You can use stress to facilitate your learning and growth | 1 = strongly disagree,  6 = strongly agree / Scale calculated: mean |
| Stress mindset | SMM-G (Crum et al., 2013) | See Crum et al. (2013) for items. | 0 = strongly disagree, 4 = strongly agree / Scale calculated: mean |
| Stress mindset | SMM-S (Crum et al., 2013) | See Crum et al. (2013) for items. | 0 = strongly disagree, 4 = strongly agree / Scale calculated: mean |
| Perceived stress | PSS-10 (Cohen & Williamson, 1988) | See Cohen and Williamson (1988) for items. | 0 = never,  4 = very often / Scale calculated: sum |
| Psychological wellbeing | WEMWBS-14 (Tennant et al., 2007) | See Tennant et al. (2007) for items. | 1 = none of the time, 5 = all of the time / Scale calculated: sum |
| Amount of stress | From (Crum et al., 2013) | Overall, how much stress do you have in your life right now? | 1 = no stress,  7 = an extreme amount of stress |
| Stressor severity appraisal | From (Crum et al., 2013) | What is the primary source of stress in your life right now? (open-ended)  How stressful do you perceive this stressor to be? | 1 = not at all stressful, 7 = an extreme amount of stress). |
| Proactive behavior | Proactive under stress scale (Keech et al., 2018) | In the last month, how often were you proactive to cope with stress?  In the last month, how often were you not proactive when under stress?*  In the last month, how often did you engage in planning your time to cope with stress?  In the last month, how often did you avoid engaging in planning your time when under stress?*  In the last month, how often did you avoid procrastination to cope with stress?  In the last month, how often did you procrastinate when under stress?* | 1 = never,  5 = very often / Scale calculated: mean |
| Physical wellbeing | HRQOL-14 Question 1 (Centers for Disease Control and Prevention., 2000) | Now thinking about your physical health, which includes physical illness and injury, for how many days during the past 30 days was your physical health not good?  During the past 30 days, for about how many days did PAIN make it hard for you to do your usual activities, such as self-care, work, or recreation? | 0 = excellent,  30 = 30 days / Scale calculated: mean; Reverse-coded for analysis to reflect healthy days |
| Trait cognitive appraisal style (challenge and threat subscales) | Cognitive Appraisal Scale (Skinner & Brewer, 2002) | See (Skinner & Brewer, 2002) for items. | 1 = strongly disagree, 6 = strongly agree/ Scale calculated: sum |
| Perceived general somatic symptoms | STICSA-T somatic subscale (Ree et al., 2008; Ree et al., 2000) | See Ree et al. (2008) for items. | 1 = almost never, 4 = almost always / Scale calculated: sum |

*Reverse-coded item

STROBE Statement—Checklist of items that should be included in reports of ***cross-sectional studies***

|  | Item No | Recommendation | Page No |
| --- | --- | --- | --- |
| **Title and abstract** | 1 | (*a*) Indicate the study’s design with a commonly used term in the title or the abstract | 2 |
|  |  | (*b*) Provide in the abstract an informative and balanced summary of what was done and what was found | 2 |
| Introduction | | | |
| Background/rationale | 2 | Explain the scientific background and rationale for the investigation being reported | 7-9 |
| Objectives | 3 | State specific objectives, including any prespecified hypotheses | 8-9 |
| Methods | | | |
| Study design | 4 | Present key elements of study design early in the paper | 13-14 |
| Setting | 5 | Describe the setting, locations, and relevant dates, including periods of recruitment, exposure, follow-up, and data collection | 13-14 |
| Participants | 6 | (*a*) Give the eligibility criteria, and the sources and methods of selection of participants | 9-10 |
| Variables | 7 | Clearly define all outcomes, exposures, predictors, potential confounders, and effect modifiers. Give diagnostic criteria, if applicable | 9-15 |
| Data sources/ measurement | 8* | For each variable of interest, give sources of data and details of methods of assessment (measurement). Describe comparability of assessment methods if there is more than one group | 10-13 |
| Bias | 9 | Describe any efforts to address potential sources of bias | N/A |
| Study size | 10 | Explain how the study size was arrived at | 13-14 |
| Quantitative variables | 11 | Explain how quantitative variables were handled in the analyses. If applicable, describe which groupings were chosen and why | 14-15 |
| Statistical methods | 12 | (*a*) Describe all statistical methods, including those used to control for confounding | 14-15 |
|  |  | (*b*) Describe any methods used to examine subgroups and interactions | N/A |
|  |  | (*c*) Explain how missing data were addressed | 16 |
|  |  | (*d*) If applicable, describe analytical methods taking account of sampling strategy | N/A |
|  |  | (*e*) Describe any sensitivity analyses | N/A |
| Results | | | |
| Participants | 13* | (a) Report numbers of individuals at each stage of study—eg numbers potentially eligible, examined for eligibility, confirmed eligible, included in the study, completing follow-up, and analysed | 9-10 |
|  |  | (b) Give reasons for non-participation at each stage | N/A |
|  |  | (c) Consider use of a flow diagram | N/A |
| Descriptive data | 14* | (a) Give characteristics of study participants (eg demographic, clinical, social) and information on exposures and potential confounders | 9-10 |
|  |  | (b) Indicate number of participants with missing data for each variable of interest | 16 |
| Outcome data | 15* | Report numbers of outcome events or summary measures | T5 |
| Main results | 16 | (*a*) Give unadjusted estimates and, if applicable, confounder-adjusted estimates and their precision (eg, 95% confidence interval). Make clear which confounders were adjusted for and why they were included | 16-19; T1-T5 |
|  |  | (*b*) Report category boundaries when continuous variables were categorized | N/A |
|  |  | (*c*) If relevant, consider translating estimates of relative risk into absolute risk for a meaningful time period | N/A |
| Other analyses | 17 | Report other analyses done—eg analyses of subgroups and interactions, and sensitivity analyses | N/A |
| Discussion | | | |
| Key results | 18 | Summarise key results with reference to study objectives | 19-20 |
| Limitations | 19 | Discuss limitations of the study, taking into account sources of potential bias or imprecision. Discuss both direction and magnitude of any potential bias | 22-23 |
| Interpretation | 20 | Give a cautious overall interpretation of results considering objectives, limitations, multiplicity of analyses, results from similar studies, and other relevant evidence | 19-22 |
| Generalisability | 21 | Discuss the generalisability (external validity) of the study results | 22 |
| Other information | | | |
| Funding | 22 | Give the source of funding and the role of the funders for the present study and, if applicable, for the original study on which the present article is based | Title page |

*Give information separately for exposed and unexposed groups.

**Note:** An Explanation and Elaboration article discusses each checklist item and gives methodological background and published examples of transparent reporting. The STROBE checklist is best used in conjunction with this article (freely available on the Web sites of PLoS Medicine at http://www.plosmedicine.org/, Annals of Internal Medicine at http://www.annals.org/, and Epidemiology at http://www.epidem.com/). Information on the STROBE Initiative is available at www.strobe-statement.org.
